# The components of Huang-Lian-Jie-Du-Decoction act synergistically to exert protective effects in a rat ischemic stroke model

**DOI:** 10.18632/oncotarget.12645

**Published:** 2016-10-13

**Authors:** Qian Zhang, Junsong Wang, Chao Zhang, Shanting Liao, Pei Li, Dingqiao Xu, Yan Lv, Minghua Yang, Lingyi Kong

**Affiliations:** ^1^ State Key Laboratory of Natural Medicines, Department of Natural Medicinal Chemistry, China Pharmaceutical University, Nanjing, 210009, P.R. China; ^2^ Center for Molecular Metabolism, Nanjing University of Science and Technology, Nanjing, 210094, P.R. China

**Keywords:** ischemic stroke, metabolomics, Huang-Lian-Jie-Du-Decoction, middle cerebral artery occlusion, oxidative stress

## Abstract

Huang-Lian-Jie-Du-Decoction (HLJDD, Oren-gedoku-to in Japanese) is commonly used in traditional Chinese medicine (TCM) to treat ischemic stroke. This study investigated the efficacy of various combinations of the major components of HLJDD, berberine (A), baicalin (B), and jasminoidin (C), on the treatment of ischemic stroke modeled by middle cerebral artery occlusion (MCAO) in rats. The effects of A, B and C individually and their combinations were investigated using proton nuclear magnetic resonance (^1^H NMR)-based metabolomics complemented with neurologic deficit scoring, infarct volume measurement, biochemistry, histopathology and immunohistochemistry, as well as quantitative real-time polymerase chain reaction (qRT-PCR) and western blotting. Ischemic stroke produces severe oxidative stress, which induces further damage. Our results show that the ABC combination treatment increased levels of cellular antioxidants that scavenged reactive oxygen species during ischemia-reperfusion via the nuclear erythroid 2-related factor 2 (Nrf2) signaling cascade. These protective effects were not observed with the other treatments. These results suggest that a combination of component herbs in HLJDD exhibit stronger effects than the individual herbs alone. Our integrated metabolomics approach also provides a tractable, powerful tool for understanding the science behind TCM formulations.

## INTRODUCTION

Stroke is one of the leading causes of mortality [1], accounting for approximately 87% of all stroke cases [2]. Unfortunately, despite of the use of aspirin and thrombolytics, there are few effective treatments for ischemic stroke due to its extremely narrow therapeutic time window [3]. Ischemic stroke is actually a series of events, including inflammation, ionic imbalance, apoptosis, angiogenesis, excitotoxicity, oxidative damage, and neurological injury, making it a huge challenge to treat with a single drug. Therefore, combinational drugs may be a rational and efficient strategy for treating ischemic stroke [4–6].

Combination therapies have been advocated by prescriptions called formulae in traditional Chinese medicine (TCM) for more than 2,500 years [7]. It is believed that, at least in some formulae, the components hit multiple targets simultaneously and have synergistic therapeutic effects [7]. However, the combinatorial rules of herbal formulae have been difficult to clarify using traditional methods due to the complexity of their components. This has hampered the use of TCM formulae, as well as the development of drugs or therapies based on these formulae. Metabolomics provides a high-throughput and holistic method to analyze disease biomarkers and assess drug efficacy [8–10]. Therefore, we sought to apply metabolomics to explore the effects of drug combinations and the underlying mechanisms of TCM formulae.

Huang-Lian-Jie-Du-Decoction (HLJDD, Oren-gedoku-to in Japanese), a famous formula of TCM, has been used to treat ischemic stroke in China and other Asian countries [11, 12]. HLJDD extracts, as well as its components, have protective effects against ischemic stroke [13–15]. Pharmacokinetic studies of the major bioactive components have been examined in a rat model of ischemic stroke, middle cerebral artery occlusion (MCAO), after oral administration of HLJDD [16–18]. Our previous studies showed that HLJDD had neuroprotective effects on rats and induced protective autophagy against the injury of cerebral ischemia/reperfusion via the MAPK-mTOR signaling pathway[19, 20]. In this study, we again used an MCAO rat model to mimic ischemic stroke and test the efficacies of three major components of HLJDD: berberine (A), baicalin (B), and jasminoidin (C), as well as their combinations, using proton nuclear magnetic resonance (^1^H NMR)-based metabolomics combined with molecular biological methods.

## RESULTS

### Therapeutic efficacy of combinations of A, B, and C

**Figure 1:**
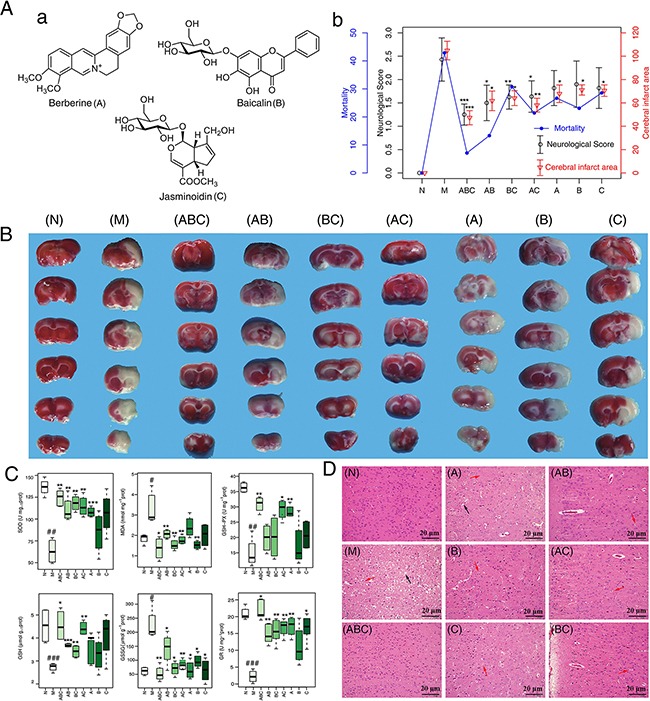
Neurological deficits, infarct volume and histopathological assessment **A.** a: The Chemical structures of berberine, baicalin and jasminoidin. b: Mortality, neurobehavioral scores and infarct volume examinations. **B.** TTC staining of brains. (n=6) **C.** Boxplots for brain tissue levels of SOD, MDA, GSH, GSSG, GSH-Px and GR in each group (n=6). At the bottom of each box, the lines drew in the box and at the top of the box represent the 1st, 2nd, and 3rd quartiles, respectively. The whiskers extended to ± 1.5 times the interquartile range (from the 1st to 3rd quartile). Outliers are shown as open circles. **D.** Histopathological examination of brain tissues by HE staining (n=6): neuronal loss and presence of numerous vacuolated spaces (black arrow), disordered neurons arrangement (red arrow). Scale bar represents 20 μm. All data are expressed as mean ± standard deviation (S.D.), n > 6. ^#^: P < 0.05, ^##^: P< 0.01 and ^###^: P< 0.001 MCAO group vs. sham group; *: P < 0.05, **: P< 0.01 and ***: P< 0.001 Drug treated groups vs. MCAO group.

Mortality, infarct volume, and neurologic score are the three most important markers for brain injury. After 24 h reperfusion, these three markers were increased in the MCAO model group compared with the control group (Figure 1A and 1B). The seven different treatment combinations all reduced the indexes of ischemia/reperfusion (I/R) rats, but the triple (ABC) combination had the best efficacy. Consistent with these observations, all treatments greatly ameliorated oxidative stress (Figure 1C), as evidenced by increased cerebral levels of superoxide dismutase (SOD), reduced glutathione (GSH), glutathione peroxidase (GSH-Px), and glutathione reductase (GR), along with decreased levels of malondialdehyde (MDA) and oxidized glutathione (GSSG). Once again, the ABC group outperformed the other combinations of A, B, and C, and rats in the ABC combination group had the least amount of brain damage (Figure 1D). MCAO rats either without treatment or with the other combinations of A, B, and C had signs of damaged neuron structure, neuronal loss, and vacuolated spaces, which were not seen in the rats that received the ABC combination treatment.

### Multivariate analysis of ^1^H NMR data

**Figure 2:**
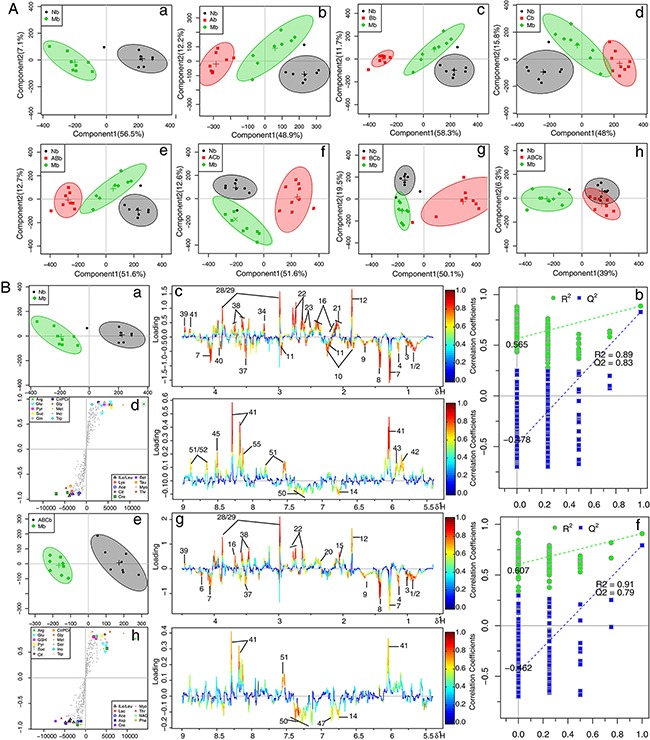
OPLS-DA analysis based on 1H NMR data from cerebral extracts of all groups **A.** Score plots according to OSC-PLS-DA analysis based on ^1^H NMR data from cerebral extracts of rats. **B.** Color-coded loading plots, S-plots, and scatter plots of the statistical validations obtained by 2000X permutation tests for OSC-PLS-DA analysis in cerebrum. a, b and c: sham vs. MCAO; d, e and f:ABC vs. MCAO.

**Figure 3:**
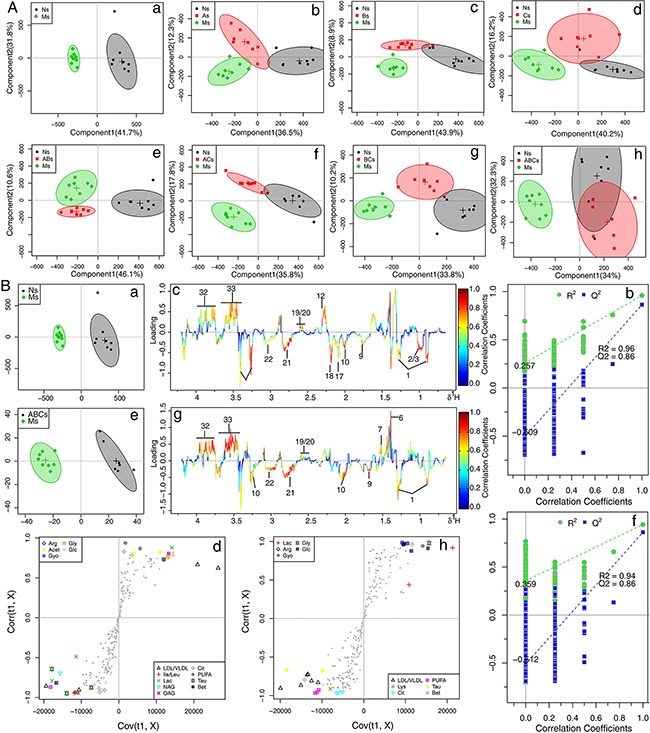
OPLS-DA analysis based on 1H NMR data from serum of all groups **A.** Score plots according to OSC-PLS-DA analysis based on ^1^H NMR data from serum of rats. **B.** Color-coded loading plots, S-plots, and scatter plots of the statistical validations obtained by 2000X permutation tests for OSC-PLS-DA analysis in serum. a, b and c: sham vs. MCAO; d, e and f: ABC vs. MCAO.

^1^H NMR data of cerebral extracts and serum from the sham, the MCAO, and A, B, and C combination groups were analyzed by orthogonal signal correction for partial least squares discriminant analysis (OSC-PLS-DA) to investigate the combinatorial effects of A, B, and C. In the score plots for cerebral extracts (Figure 2Aa-g), the showcased clusters correspond to metabolic patterns in different groups with each point representing one sample. Interestingly, the metabolic patterns in rats treated with combinations consisting of one or two components were quite different from those treated with the ABC regimen. The three single treatments of A, B, and C, and the two combinations of AB were well separated from the control group with the MCAO model group in between. AC and BC combination groups were also clearly separated from the model and control groups, between which, no separation was achieved along the first component, reflecting the bias of single therapies. The ABC treatment group and sham group overlapped with each other, and both were separated from the MCAO group (Figure 2Ah), highlighting the ability of the ABC combination treatment to protect against metabolic disturbance following MCAO injury. Similarly, in the OSC-PLS-DA score plots for serum (Figure 3A), individual drug and combination treatment groups were located between MCAO and the sham group, with the ABC group closest to and overlapping with the sham group, again indicating that the ABC combination yields synergy in the treatment of MCAO rats.

### Metabolic alterations in MCAO and ABC-treated rats

To further explore the metabolic perturbations induced by MCAO, the NMR data of the MCAO group were directly compared with the sham and ABC-treated group by OSC-PLS-DA analysis. The score plots for cerebrums and serum both presented a clear clustering of the sham and MCAO groups (Figure 2Ba and Figure 3Ba) with a well goodness of fit (R^2^Y = 0.89, Q^2^Y = 0.83 for cerebrum, Figure 2Bb; R^2^Y = 0.96, Q^2^Y = 0.86 for serum, Figure 3Bb), indicating that the models were good. Metabolites that contributed to group separation were then visualized and color-coded according to the absolute correlation coefficient of each variable with each group. A hot-colored signal (red) indicated a more significant contribution to class separation than a cold-colored one (blue), and is presented in a covariance-based pseudo-spectrum [21]. S-plots were also used to identify altered metabolites, which are located in the upper right or lower left quadrant and farther away from the origin. The color-coded loading plots (Figure 2Bc and Figure 3Bc) and S-plots (Figure 2Bd and Figure 3Bd) showed clear increases of leucine, isoleucine, lactate, alanine, lysine, taurine, betaine, acetoacetate, creatinine, GABA, AMP, glutamate, glutamine. and threonine, and significant decreases of glucose, NADPH, acetate, pyruvate, citrate, isocitrate, TMAO, glycine, glycerol, aspartate, ascorbic acid, myo-inositol, adenosine, inosine, nicotinurate, GSH, uridine and uracil in the MCAO group as compared with the sham group. Clear separation was achieved between samples obtained from the MCAO and ABC groups for both cerebrum (Figure 2Be) and serum measures (Figure 3Be), as evidenced by high Q^2^ values (Figure 2Bf and Figure 3Bf). OSC-PLS-DA loading plots (Figure 2Bg and Figure 3Bg) and S-plots (Figure 2Bh and Figure 3Bh) revealed that ABC treatment ameliorated the metabolic disturbance in cerebrums and serum resulting from MCAO.

### Univariate analysis of ^1^H NMR data

**Figure 4:**
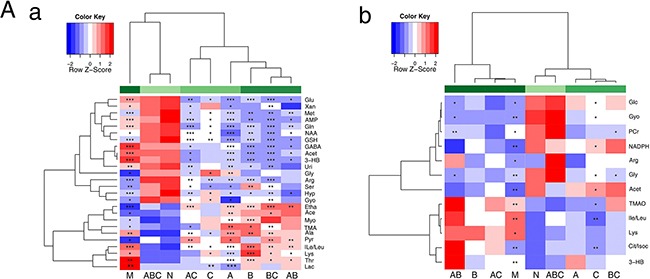
Heatmap visualization of the metabolites in cerebral extracts and serum Heatmap visualization of the z-scored levels of metabolites in cerebral extracts **A.** and serum **B.** with stars denoting the significance. Rows represent metabolites and columns represent groups. All groups were compared with the sham group (N). Color key indicates metabolite quantities, white: no significant change, deep red: highest, deep blue: lowest. * P< 0.05, ** P< 0.01 and *** P< 0.001.

Altered metabolites were further tested for their between-group differences using univariate analysis, which was visualized in heatmaps (Figure 4). As shown in Figure 4A, imbalanced metabolites in the cerebrum of MCAO rats were still present in rats treated with combinations consisting of one or two of the components, and these groups were also different from the sham group. In contrast, there was no statistical difference between the ABC treatment group and sham group, which were clustered together. Consistent with these observations, the ABC combination treatment outperformed the individual use of A, B, or C or the combination of two treatments, and clustered together with the sham group, demonstrating the synergistic benefit of ABC combination treatment (Figure 4B).

### Synergistic effects of ABC treatment on the expression of markers related to ischemic stroke

**Figure 5:**
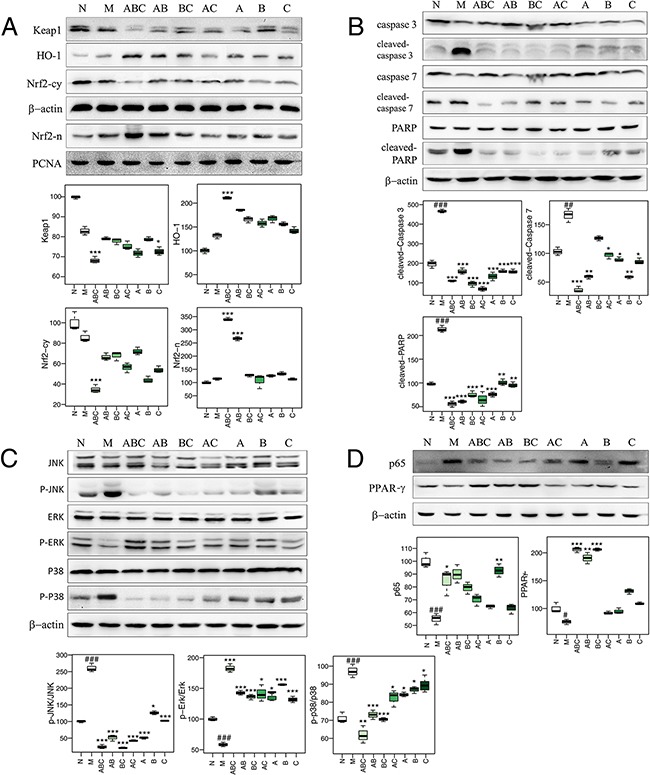
Protein expression as determined by western blotting **A.** The expression of cytosolic Nrf2 (Nrf2-cy), HO-1, Keap1 and nuclear Nrf2 (Nrf2-n) in cerebrums treated with various combinations of A, B, and C. (n=6) **B.** The expression of caspase 3, cleaved caspase 3, caspase 7, cleaved caspase 7, PARP, and cleaved PARP in cerebrums treated with various combinations of A, B, and C. (n=6) **C.** The expression of p38, photo-p38 (p-p38), ERK, phospho-ERK (p-ERK), JNK, and p-JNK (phospho-JNK) in cerebrums treated with various combinations of A, B, and C. (n=6) **D.** The expression of p65 and PPAR-γ in cerebrums treated with various combinations of A, B, and C. (n=6)

Oxidative stress and inflammation cause I/R-induced cellular apoptosis and neuronal injury [22]. Both biochemical assays and metabolomics data suggested that the ABC combination treatment ameliorated oxidative stress and blocked perturbations to glutathione metabolism in MCAO rats. Therefore, we next investigated the effects of ABC combination on oxidative stress-related pathways. Compared with the sham group, the MCAO group had slight (but not statistically significant) increases in the expression of nuclear erythroid 2-related factor 2 (Nrf2) and cytoplasmic heme oxygenase-1 (HO-1), which were both increased by ABC treatment. Similarly, the expression of the Nrf2 inhibitor, Keap-1, in the cytosolic fraction was slightly decreased in the MCAO group, and significantly decreased by ABC treatment (Figure 5A). The expression of several oxidative stress-related protein kinases, such as phospho-p38 and phospho-JNK, were increased in the MCAO group and were decreased by ABC treatment (Figure 5B). A decrease in the expression of phospho-ERK was also found in the MCAO group, while its expression increased with ABC treatment.

**Figure 6:**
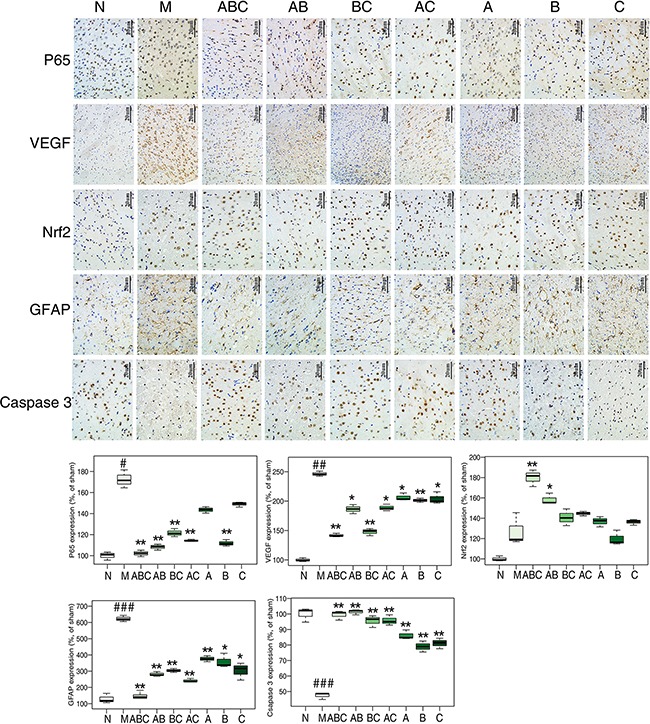
Proteins expression determined by immunohistochemical staining **A.** The expression of p65, VEGF, Nrf2, GFAP, and caspase 3 in cerebral tissues of rats treated with various combinations of A, B, and C as determined by immunohistochemical staining (n=6). Scale bar represents 20 μm.

**Figure 7:**
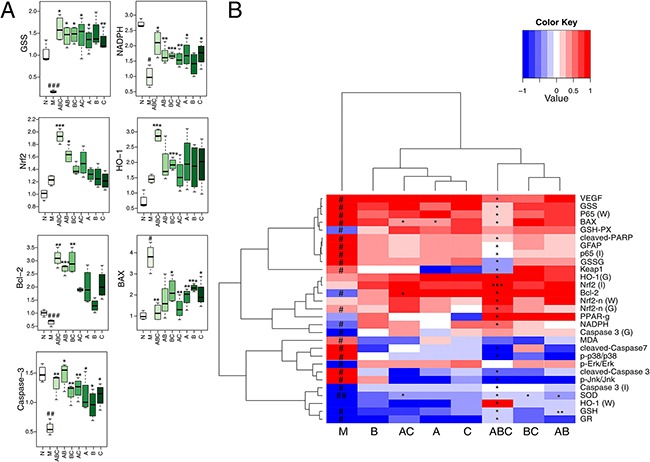
mRNA expression and heatmap of all measured parameters **A.** Boxplots for the mRNA expression analysis by RT-PCR in cerebrums. (n=6) **B.** The heatmap of all the parameters determined by biochemistry, immunohistochemistry (I), qRT-PCR (G), and western blotting (W). Results of quantitative analysis values are expressed as mean ± SD (n = 5). ^#^: P < 0.05, ^##^: P< 0.01 and ^###^: P< 0.001 MCAO group vs. sham group; *: P < 0.05, **: P< 0.01 and ***: P< 0.001 Drug treated groups vs. MCAO group.

Markers of inflammation (NF-κB-p65 [23], PPAR-γ [24]), apoptosis (caspase proteases [25], Bcl-2, Bax [26]), neurotrophic factors (VEGF [27], GFAP [28]), nicotinamide adenine dinucleotide phosphate (NADPH) and glutathione synthetase (GSS) were also measured by western blotting (Figure 5C and 5D), immunohistochemistry (Figure 6), and qRT-PCR (Figure 7A), and visualized in a heatmap (Figure 7B). Compared with the sham rats, MCAO rats had increased expression of NF-κB-p65, cleaved-caspase-3/7, cleaved-PARP, Bax, VEGF, GFAP and GSS, and decreased expression of PPAR-γ, caspase-3/7, PARP, Bcl-2 and NADPH. ABC treatment reversed all of these changes in expression, but A, B or, C alone or in double combinations only modulated a fraction of these parameters.

### Metabolite pathway analysis

**Figure 8:**
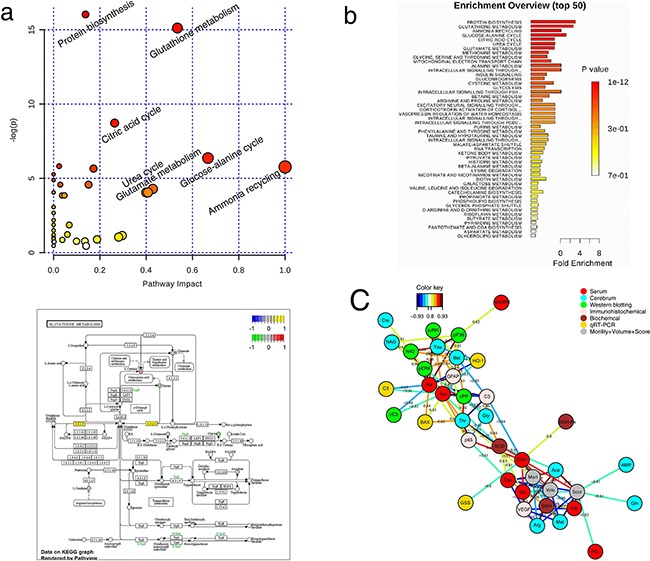
Correlation network of differential metabolites in cerebral extracts and serum of all groups **A.** Disturbed metabolic pathway in MCAO rats as visualized by a: bubble plots and b: Enrichment Overview. **B.** Visualization of glutathione metabolism pathway relevant for I/R by KEGG. **C.** Correlation network determined by canonical (sparse-partial least squares, sPLS) analysis using metabolite concentrations as X variables and other parameters as Y variables. The network is graphically represented with metabolites and parameters as nodes, and correlations above a threshold (0.8) as edges (color coded according to the correlation coefficients, bluish for negative and reddish for positive correlations).

Metabolites selected based on OSC-PLS-DA loadings/S-plots were subjected to pathway analysis using MetPA (http://www.metaboanalyst.ca) and KEGG (http://www.genome.jp/kegg/) to identify biologically meaningful metabolic patterns and relevant pathways (Figure 8A and Figure 8B). Glutathione metabolism accounted for a large proportion of the pathway alterations induced by I/R. Canonical regression analysis (sparse-partial least-squares, sPLS) was performed with metabolite concentrations as X variables and the other parameters as Y variables to assess the relationships among gene expression, protein expression, biochemical parameters, immunohistochemical parameters, mortality, infract volume and neurological score (Figure 8C). Among these parameters, most of the significant correlations were associated with oxidative stress. SOD was located in the center of the network and was positively correlated with GSH and GSH-Px, indicating that oxidative stress might have occurred in MCAO rats.

## DISCUSSION

In this study, a ^1^H NMR-based metabolomics approach was adopted and complemented with current biochemical techniques to investigate the synergistic effects of the major active components of HLJDD and explore their underlying mechanisms. The full combination of berberine (A), baicalin (B), and jasminoidin (C) had the best efficacy among all treatments according to various indexes such as neurologic deficit score, infarct volume, histopathology, biochemistry, immunohistochemistry, and molecular markers. Consistent with these results, OSC-PLS-DA analysis of NMR metabolic profiles suggested synergic effects of ABC on the treatment of MCAO. These results therefore justify the rationale behind the TCM formula for HLJDD and show that several weak-effect component herbs can be combined to exhibit strong effects.

Metabolic pathway analysis revealed that MCAO severely perturbed glutathione metabolism, suggesting that oxidative stress was induced by ischemic stroke. Restoration of the blood supply by reperfusion after ischemia brings oxygen and glucose to neurons in an amount exceeding their normal consumption, resulting in an increased supply of oxygen[29, 30]. Excessive oxygen destroys the balance between pro-oxidants and antioxidants, producing excessive free radicals and ROS, which are responsible for a series of pathological reactions in I/R stroke [29, 31, 32].

Oxidative damage evokes lipid peroxidation, protein degradation, and DNA lesions, leading to severe cell injury and even cell death[31, 33, 34]. Cell membrane lipids, rich in polyunsaturated fatty acids, are especially susceptible to oxidative damage [35], as evidenced by the increased levels of MDA (the final product of poly unsaturated fatty acids peroxidation [36]) in cerebral tissues of MCAO rats. The elevated levels of amino acids (leucine, isoleucine, valine, alanine, threonine and lysine) in serum and cerebral extracts suggests accelerated protein degradation by ROS [37], and increased levels of uridine indicates DNA damage was induced by oxygen radicals, aggravating the I/R injury [38].

The oxidative status of MCAO rats could also be reflected by antioxidant enzymes, such as SOD, GSH-Px, and GSH. Compared with sham rats, the activity of SOD was significantly decreased in MCAO rats. The GSH redox system is another important antioxidant mechanism of the body. Under the catalysis of GSH-Px, GSH is oxidized to disulfide (GSSG) and GSSG is converted back to GSH by GR in a NADPH-dependent manner [39, 40]. Compared with the sham-operated rats, levels of GSH-Px, GR, and GSH were decreased, accompanied by the increase of GSSG in MCAO rats, demonstrating the overconsumption of GSH and as a result, the accumulation of GSSG. To replenish GSH during I/R, the body has to facilitate its synthesis, as evidenced by the decreased levels of its precursors, glycine and glutamate, and the increased expression of the *Gss* gene in MCAO rats.

The disturbances in GSH metabolism and oxidative stress in MCAO rats were still severe with treatment of A, B, or C alone, or with the combination of two of these components, but were greatly ameliorated after ABC treatment, demonstrating the synergistic benefit of the ABC combination. Nrf2, a master regulator of anti-oxidative defense responses, was investigated to further clarify the protective mechanism of ABC combination treatment. Nrf2 is a basic leucine zipper transcription factor that stimulates the expression of numerous ROS detoxifying and antioxidant genes [41, 42]. Under normal conditions, Nrf2 localizes in the cytoplasm where it interacts with the actin binding protein, Keap1, and is rapidly degraded by the ubiquitin-proteasome pathway. This quenching interaction maintains low basal expression of Nrf2-regulated genes. In contrast, chemical signals imparted by ROS or electrophilic insults (eg. GSH) target the Nrf2-Keap1 complex, dissociating Nrf2 from Keap1. Stabilized Nrf2 then translocates to the nucleus, where it dimerizes with small Maf proteins and binds to the DNA at the antioxidant response elements (ARE) [43] to activate transcription of Nrf2 target genes [44–46], including HO-1 and other phase II antioxidant enzymes. The modulation of HO-1 might therefore provide important endogenous defenses against oxidative injury in the brain during ischemia and inflammation [47–49]. In this study, ABC treatment decreased the expression of Keap-1 in the cytosol and induced the translocation of Nrf2 to the nucleus. This was accompanied by increases in both mRNA and protein levels of HO-1, thus enhancing the cellular antioxidant defense system. These results indicate that the activation of Nrf2 signaling pathway may underlie the protective effects of ABC treatment.

Previous studies have shown that the upregulation of Nrf2 is associated with its accumulation in the nucleus, due either to the degradation of Keap1 or from the phosphorylation of Nrf2 by protein kinases, such as, p38, ERK and JNK [50]. Our experiments revealed that ABC treatment activated ERK signaling and inhibited p38 and JNK phosphorylation in MCAO rats. We may therefore presume that ABC-induced Nrf2 expression could be ascribed to MAPK pathways, thus potentiating the effects of ABC during treatment. These findings demonstrate that ABC treatment increases cellular antioxidants to scavenge over-generated ROS during I/R via the Nrf2 signaling cascade. Whether ABC alters Keap1 and/or Nrf2 directly or through upstream signaling (i.e. MAPK) requires further study.

**Figure 9:**
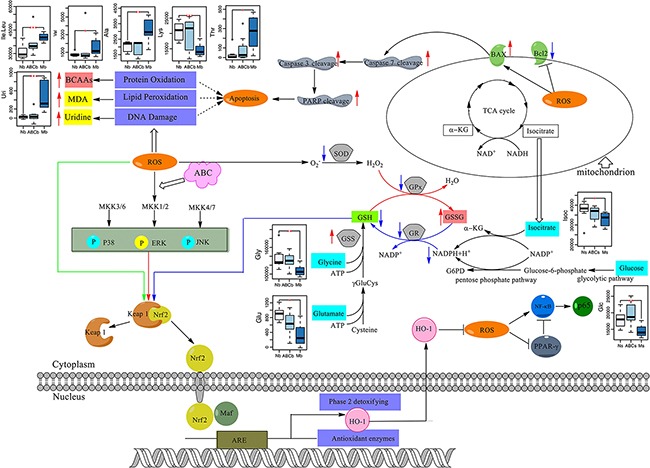
The signaling pathway changes triggered by ABC combination treatment Red arrows represent the increased metabolites in MCAO rats and blue arrows represent the decreased metabolites in MCAO rats, as measured by ^1^H NMR. ABC combination treatment greatly increased cellular antioxidants to scavenge over-generated ROS during I/R.

Based on our findings, we propose a model of the synergistic actions of HLJDD components as protection against ischemic stroke in an MCAO rat model (Figure 9). Under this model, ABC treatment alters cell metabolism and has neuroprotective effects by inhibiting ROS formation and alleviating oxidative stress. ABC treatment inactivates Nrf2 either by regulation of Keap1 or ERK signaling pathways. HO-1 is downstream of the Nrf2-active signaling pathway, and as a result, ROS production is inhibited and oxidative stress is attenuated.

In summary, MCAO rats suffered from the over-generation of ROS during I/R and developed severe neuronal losses and neurological deficits. ABC combination treatment outperformed individual use of A, B, and C or the combination of two of them, in the treatment of ischemic stroke, demonstrating a favorable synergistic effect of the three major components of HLJDD. Using an integrated metabolomics approach combined with molecular biology gave an added benefit in assessing the drug combination effects and dissecting their complicated mechanisms. Thus, this method is a feasible and powerful tool that can be used to increase our understanding of the ancient wisdoms of TCM formulating principles.

## MATERIALS AND METHODS

### Chemicals and reagents

Berberine (purity ≥ 98.0 %) and jasminoidin (purity ≥ 98.0 %) were purchased from Qingdao Jieshikang Biotech Co., Ltd. (Qingdao, China), and baicalin (purity ≥ 98.0%) was purchased from Dalian Meilun Biotech Co., Ltd. (Dalian, China).

Sodium 3-trimethylsilyl-1-(2,2,3,3-2H_4_) propionate (TSP) was obtained from Sigma (St. Louis, Mo, USA). Deuterium oxide (D_2_O, 99.9 %) was purchased from Sea Sky Bio Technology Co. Ltd. (Beijing, China). Chloral hydrate and acetonitrile were obtained from Sinopharm Chemical Reagent Co., Ltd. (Shanghai, China). Ultra-pure distilled water, prepared from a Milli-Q purification system, was used. Cleaved caspase-3 (Asp175) Rabbit mAb, procaspase-3 Antibody, cleaved caspase-7 (Asp198) Antibody, procaspase-7 Antibody, cleaved PARP (Asp214) Antibody, PARP Antibody, p65 Antibody, PPAR-γ Antibody, phospho-ERK (Thr202/Thr204) Rabbit mAb, phospho-p38 (Thr180/Thr182) Rabbit mAb, phospho-JNK (Thr183/Thr185) Rabbit mAb, ERK Rabbit mAb, JNK Rabbit mAb, p38 MAP Kinase Antibody, β-actin Mouse mAb, PCNA Rabbit mAb, anti-rabbit IgG antibody, anti-mouse IgG antibody were purchased from Cell Signaling Technology (Beverly, MA, USA).

### Animal procedures

Male Sprague-Dawley rats (250 ± 20 g) were obtained from the Laboratory Animal Research Center, Nanjing University (Nanjing, China). Rats were housed in an air-conditioned room at constant room temperature (25 ± 2 °C) and air humidity (50 ± 10 %), with a 12-hour light and dark cycle, with free access to water and rodent chow (5 rats per cage). All procedures were approved by the Institutional Animal Care and Use Committee (IACUC) of China Pharmaceutical University.

Rats were randomly divided into nine groups (n ≥ 20): sham operation (N), MCAO model (M), and treatment with berberine (A), baicalin (B), andjasminoidin (C), or their combinations (AB, AC, BC, and ABC; Supplementary Table S1). The optimized dosage of 20 mg/kg body weight (BW) was used, according to the results of preliminary experiments (data not shown). Drugs were combined according to their ratios in the HLJDD formula: berberine 5.05%, baicalin 4.02% and jasminoidin 2.70% (Figure 1A) [51]. Drugs were dissolved in 0.5% carboxymethyl cellulose sodium salt (CMC-Na) and intragastrically (i.g.) administered to rats (20 mg/kg BW) once a day for 7 days, while the sham and MCAO groups were administered equivalent amounts of 0.5% CMC-Na for 1 week.

### Middle cerebral artery occlusion (MCAO) model in rats

MCAO surgery was performed as our previously described [52]. Briefly, animals were anesthetized with chloral hydrate (3.5%, 350 mg/kg, i.p.). The right common carotid artery (CCA), the right external carotid artery (ECA), and the right internal carotid artery (ICA) were exposed and isolated from connective tissues. A 4-0 mono-filament nylon suture (Beijing Sunbio Biotech Co., Ltd., Beijing, China) with a rounded tip was inserted into the ICA through the ECA stump and gently advanced to occlude the CCA. After 2 h sustained ischemia, reperfusion was performed by the withdrawal of the inserted filament. Sham-operated rats received the same surgical procedures except that the arteries were not occluded. All surgical operations were done in a sterile environment with body temperatures maintained at 37 ± 0.5°C until neurological evaluation. A laser Doppler flowmeter (FLPI2, Moor Instruments Ltd., Axminster, UK) was used to confirm that the regional cerebral blood flow (rCBF) had decreased to 30% of pre-ischemia levels immediately after the occlusion to. Animals whose blood flow decreased to 30% of pre-ischemia levels were used for further study (Supplementary Figure S1).

### Neurological deficit evaluation

Twenty-four hours after reperfusion, rats were tested for sensorimotor performance by observers blind to the experiment using Longa's five-point neurological severity scale before sacrifice [53]. Neurological scores were graded by the following scale: 0, no neurological deficit; 1, failure to extend right forelimb; 2, circling to the contralateral side; 3, falling to the contralateral side at rest; and 4, no spontaneous motor activity.

### Infarct volume measurement

Brain infarct size was evaluated by 2,3,5-triphenylte trazolium chloride (TTC) staining method [54]. Rats were decapitated after neurological examination. The brains were removed, sectioned into six 2 mm-thick coronal slices, and then stained with 0.5% TTC in 0.1 M PBS (pH 7.4) for 30 min at 37°C. Sections stained with TTC were photographed and analyzed by image analysis software (Image-Pro Plus 6.0).

### Biochemical assay

After 24 h of reperfusion, rats were sacrificed and brains were removed. The homogenates extracted from the cortex of the ischemic brain hemispheres were analyzed for biochemical parameters. The levels of superoxide dismutase (SOD), glutathione peroxidase (GSH-PX), glutathione (GSH), glutathione disulfide (GSSG), glutathione reductase (GR), and malondialdehyde (MDA) were measured using commercially available kits (Nanjing Jianchen Biotech Inc., China).

### Histopathological and immunohistochemical examination

For histological examination, brain tissues were quickly removed, rinsed with cold phosphate buffered saline (PBS), and then immediately fixed in 10% neutral-buffered formaldehyde for 48 h. After fixation, tissues were embedded in paraffin, sliced to 5 μm thickness, and then stained with hematoxylin and eosin (H&E) for microscopic observation. The histopathology results were evaluated by prof. Ning Su (Southeast University, Nanjing, China) who was blinded to the experiments. For immunohistochemical examination, sections of formalin-fixed, paraffin embedded brain tissues were used and the activity of caspase-3, NF-κB (Nuclear factor-kappa B)-p65, nuclear factor erythroid 2-related factor 2 (Nrf2), glial fibrillary acidic protein (GFAP) and vascular endothelial growth factor (VEGF) were evaluated by Goodbio technology CO., LTD (Nanjing, China). Staining was photographed under light microscopy, and analyzed by image analysis software (Image-Pro Plus 6.0).

### Sample preparation and ^1^H NMR analysis

Frozen ischemic hemispheres (200-300 mg) were homogenized in ice-cold solvent (50% acetonitrile/H2O, v/v, 5 ml g^-1^ tissue), vortexed, and then centrifuged at 12,000 g for 10 min at 4°C. The supernatant was collected and concentrated under a stream of nitrogen and lyophilized. Dried cerebrum extracts were reconstituted in 600 μL D_2_O phosphate buffer (0.2 mol L^-1^ Na_2_HPO_4_ and 0.2 mol L^-1^ NaH_2_PO_4_, pH 7.4, containing 0.05% TSP). TSP acted as a chemical shift reference (δ 0.0), D_2_O provided a lock signal and phosphate buffer was added to minimize NMR shift variation due to pH discrepancy. For serum samples, 300 μL of each sample was added to 300 μL D_2_O phosphate buffer. After vortexing and centrifugation at 12,000 g for 10 min at 4°C to remove residues, the transparent supernatant solution was pipetted into 5 mm NMR tube for NMR analysis.

^1^H NMR spectra were recorded at 25 °C on a Bruker AV 500 MHz spectrometer. For tissue samples, a nuclear overhauser enhancement spectroscopy (NOESY) pulse sequence (relaxation delay-90°-μs-90°-tm-90°-acquire-FID) was used to suppress the residual water signal. For serum samples, the transverse relaxation-edited Carr-Purcell-Meiboom-Gill (CPMG) spin-echo pulse sequence (RD-90°-(τ-180°-τ) n-ACQ) with a total spin-echo delay (2 nτ) of 40 ms was used to suppress broad signals from any residual macromolecules (i.e., proteins or lipoproteins). ^1^H NMR spectra were collected with 128 transients into 32,768 (32 K) data points over a spectral width of 10000 Hz, with an acquisition time of 3.27 s and a relaxation delay of 3.0 s. The free induction decays (FIDs) were weighted by an exponential window function with a 0.3 Hz line-broadening factor prior to Fourier transformation.

### Spectral pre-processing and data analysis

The processing methods used on the raw NMR data were based on protocols described in our previous work [55]. Briefly, the spectra for all samples were manually phased and baseline corrected, and referenced to TSP at 0.0 ppm using Bruker Topspin 3.0 software (Bruker GmbH, Karlsruhe, Germany), automatically exported to ASCII files using MestReNova (Version 8.0.1, Mestrelab Research SL, Santiago de Compostela, Spain), and then imported into “R” (http://cran.r-project.org/) for multivariate data analysis with an in-house developed R-script. The one-dimensional (1D) spectra were segmented into 0.0025 ppm integrated spectral buckets between 0.2 and 10 ppm for statistical analysis. The region of residual water and affected signals (4.65-5.25 for cerebrum extracts, and 4.70-9.70 for serum) was excluded. To account for different sample dilutions sample dilution effects, all binned spectra were probability quotient normalized, and subsequently mean-centered and pareto-scaled before further multivariate analysis.

Principal component analysis (PCA) and orthogonal signal correction partial least-squares discriminant analysis (OSC-PLS-DA) were applied to NMR data. PCA is an exploratory unsupervised pattern recognition method that transforms the variables in a data set into a smaller number of new latent variables, called principal components (PCs) [56]. However, no obvious clustering was observed when variables were not selected (data not shown). PLS-DA is a supervised extension of PCA to find components of independent variable space that are relevant to the outcome space [57]. OSC is a filtering method to minimize the influence of unrelated signals, i.e. systematic variation or noise [57]. It is important to avoid over fitting after OSC treatment, to prevent poor predictive performance; hence, a precise determination of the number of removed OSC factors is very important and here only one factor was removed. All OSC-PLS-DA models were validated by a repeated two-fold cross-validation and permutation test (2000 times) [58]. R^2^ and Q^2^ parameters indicated the goodness of fit and the ability of prediction, respectively. Color-coded loadings plot and S-plot were constructed to reveal variables that contributed to the group separation. The fold-change values of metabolites and their associated p-values corrected by Benjamini & Hochberg-adjusted method [59] were calculated and visualized in heatmaps.

### Metabolites identified in ^1^H NMR spectra of serum and tissue

Representative 500 MHz ^1^H NMR spectra of serum and cerebrum samples from the sham, the MCAO, the ABC-treated rats were shown in Supplementary Figure S2 with the assignment of metabolites. The signals were assigned by querying publicly accessible metabolomics databases [60], such as Madison (http://mmcd.nmrfam.wisc.edu/), MMCD (http://mmcd.nmrfam.wisc.edu/), ECMDB (http://www.ecmdb.ca/) and HMDB (http://www.hmdb.ca/), aided by Chenomx NMR suite 7.5 (Chenomx Inc., Edmonton, Canada) and the statistical total correlation spectroscopy (STOCSY) analysis method. The detailed information of the metabolites are listed in Supplementary Tables S2 and S3.

### STOCSY

2D-STOCSY was used to identify correlations between spectral resonances of interest to assist in metabolite identification. Resonances arising from the same molecules are highly correlated (correlation coefficient *r* = 1 theoretically), which could help the elucidation of metabolites and resolve the ambiguous peaks due to overlapping. For example, strong correlations between *δ* 2.14 (m) and *δ* 2.44 (t) helped the assignment of glutamine, and the correlation between the signal at 2.10 ppm to that at 2.34 ppm made the assignments of them to glutamate (Supplementary Figure S3).

### Real-time quantitative RT-PCR

Total RNA was extracted from ischemic cortical tissue using RNAiso Plus reagent (TaKaRa Biotechnology Co., Ltd, Dalian, China) following the manufacturer's protocol. Quantitative real-time polymerase chain reaction (qRT-PCR) was performed with a LightCycler 480 (Roche Molecular Biochemicals, Mannheim, Germany). Transcript levels were quantified by using the 2^−ΔΔ*CT*^ method [61]. The relative gene expression level of each gene was normalized to that of *Actb*. Forward and reverse primers used in the present study are listed in Supplementary Table S4.

### Western blot analysis

Western blot analysis was performed on ischemic cortical samples. Briefly, samples were homogenized in 1X RIPA lysis buffer (50 mM Tris-HCl, pH 7.4, 150 mM NaCl, 0.25% deoxycholic acid, 1% NP-40, 1 mM EDTA, and phosphatase and protease inhibitors) (Amresco, Solon, USA) to extract the total proteins. Cytosolic and nuclear extracts were performed using a cytosolic/nuclei isolation kit (Beyotime Biotechnology Co., Ltd., Nanjing, China), according to the manufacturer's protocols. Protein concentration was determined using a bicinchoninic acid protein (BCA) assay kit (Beyotime, Haimen, China). Equal amounts of protein were separated by sodium dodecyl sulfate (10%, 12% or 15%) polyacrylamide gel electrophoresis (SDS-PAGE, BioRad Laboratories, Hercules, CA), wet-transferred to PVDF membrane (BioRad Laboratories, Hercules, CA) and blotted with primary antibodies specific for Nrf2, Kelch-like ECH associated protein 1 (Keap1), heme oxygenase-1 (HO-1), NF-κB-p65, peroxisome proliferator-activated receptor gamma (PPAR-γ), PCNA, cleaved caspase-3, caspase-3, PARP, cleaved PARP, caspase-7, cleaved caspase-7, p38 mitogen-activated protein kinase (p38), extracellular signal-regulated kinase (ERK), and c-Jun N-terminal kinase (JNK), phospho-ERK, phospho-p38, phospho-JNK, β-actin, and PCNA, then probed with secondary isotype specific antibodies tagged with horseradish peroxidase (Cell Signaling Technology). Bound immuno-complexes were detected using ChemiDOC™ XRS+ system (BioRad Laboratories, Hercules, CA).

### Statistical analysis

Assays were conducted at least three times unless otherwise stated. All data, except for mortality were expressed as mean ± standard deviation (S.D.) and were compared using a one-way analysis of variance (ANOVA) followed by Tukey's multiple-comparison test. A p value less than 0.05 was considered as statistically significant.

## SUPPLEMENTARY MATERIALS FIGURES AND TABLES


