# Supplementary material for: The components of Huang-Lian-Jie-Du-Decoction act synergistically to exert protective effects in a rat ischemic stroke model
Source: Oncotarget. 2016 Oct 13;7(49):80872–87. doi: 10.18632/oncotarget.12645 (PMC5348361; doi:10.18632/oncotarget.12645)
Supplement: Supplementary file 1 [file oncotarget-07-80872-s001.pdf]

The components of Huang-Lian-Jie-Du-Decoction act synergistically to exert protective effects in a rat ischemic stroke model

SUPPLEMENTARY FIGURES AND TABLES

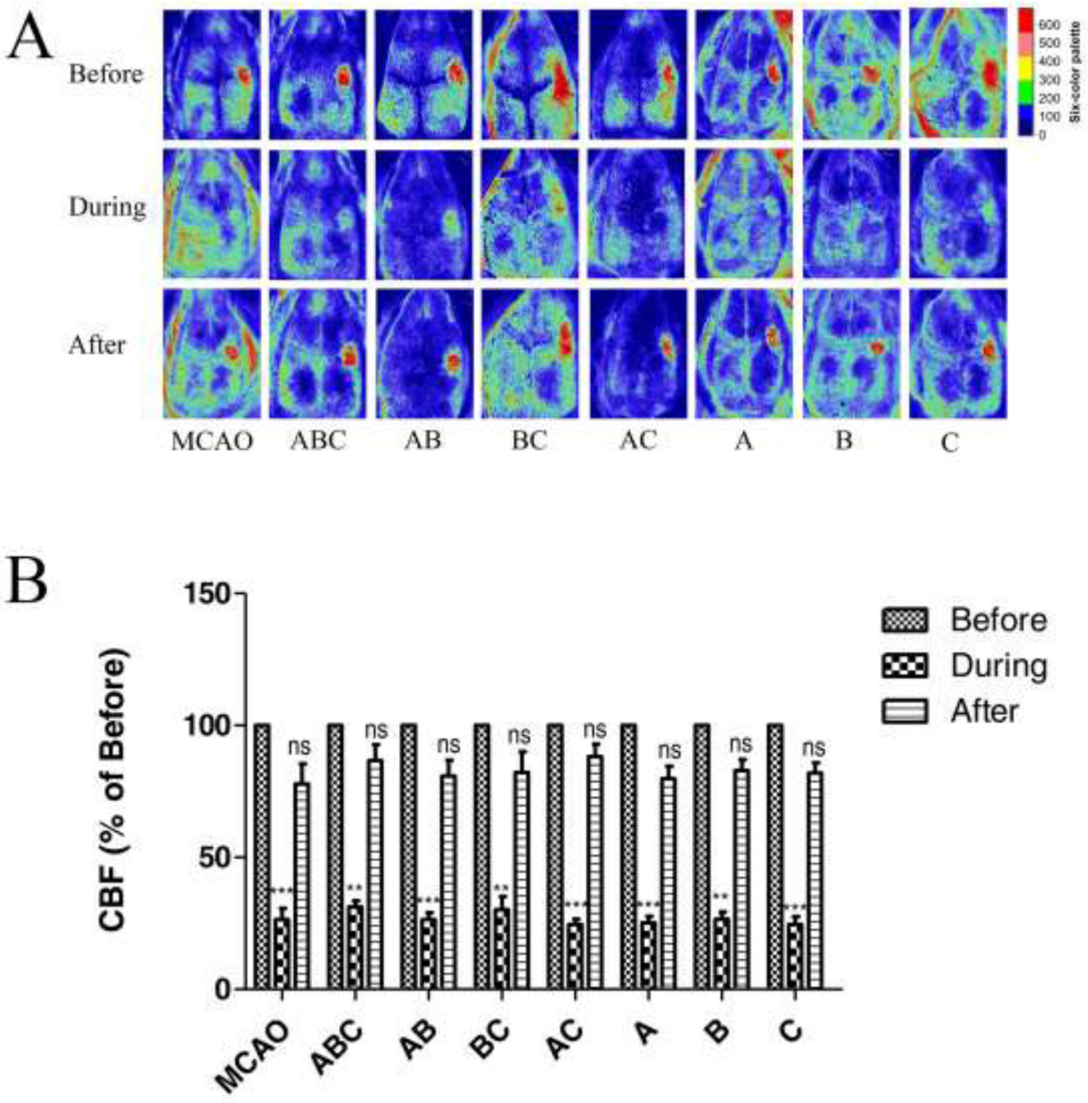

Supplementary Figure S1: A. The representative images of regional cerebral blood flow (rCBF) of ipsilateral cortex in model rats before, during and after ischemia, each column representing a rat. The magnitude of rCBF is denoted by different colors, from blueish to redish representing ascending values of rCBF. **B.** Quantitative analysis of rCBF in different groups. Data are expressed as mean ± SD, n=6. * P<0.05 vs pre-ischemia levels, ** P<0.01 vs pre-ischemia levels, *** P<0.001 vs pre-ischemia levels.

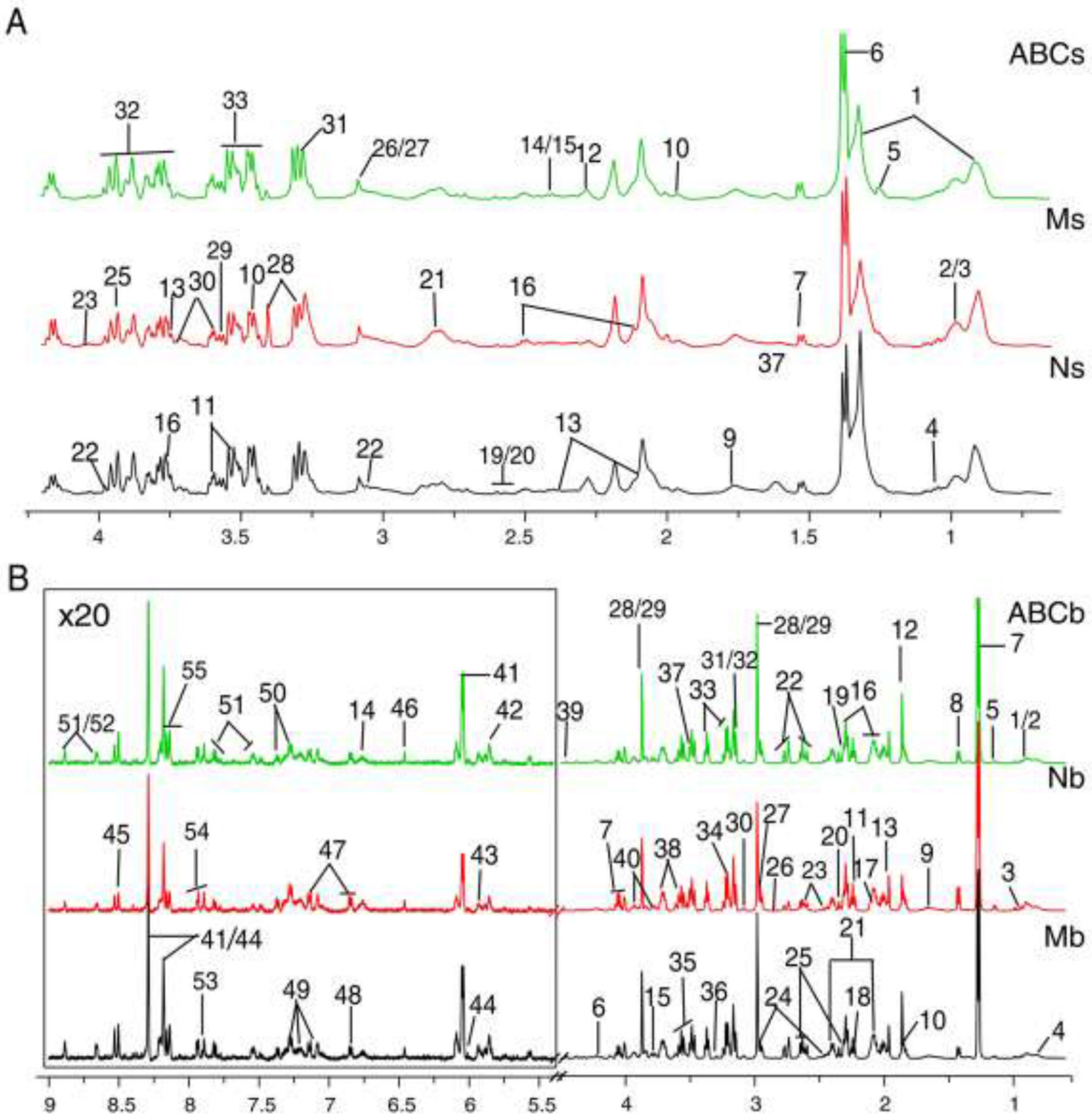

Supplementary Figure S2: Typical 500 MHz ^{1}H NMR spectra of serum **A.** and cerebrum **B.** obtained from the sham, the MCAO and the ABC-treated groups. Metabolites in serum: 1, Low density lipoprotein/very low density lipoprotein (LDL/VLDL); 2, Valine (Val); 3, Leucine (Leu); 4, Isoleucine (Ile); 5, β-Hydroxybutyrate (3-HB); 6, Lactate (Lac); 7, Alanine (Ala); 8, Lysine (Lys); 9, Arginine (Arg); 10, Myo-Inositol (Myo); 11, Acetoacetate (Acet); 12, Glutamate (Glu); 13, Pyruvate (Pyr); 14, Succinate (Suc); 15, Glutamine (Gln); 16, Citrate (Cit); 17, Isocitrate (Isoc); 18, Nicotinamide adenine dinucleotide phosphate (NADPH); 19, Cystine (Cys); 20, Creatinine (Cre); 21, Creatine (Cr); 22, Phosphocreatine (PCr); 23, Trimetlylamine oxide (TMAO); 24, Taurine (Tau); 25, Glycine (Gly); 26, Glycerol (Gyo); 27, Betaine (Bet); 28, α-Glucose (α-Glc); 29, β-Glucose (β-Glc). Metabolites in cerebrum tissue: 1, Isoleucine (Ile); 2, Leucine (Leu); 3, Valine (Val); 4, 2-Hydroxybutyrate (2-HB) 5, β-Hydroxybutyrate (3-HB); 6, Threonine (Thr); 7, Lactate (Lac); 8, Alanine (Ala); 9, Lysine (Lys); 10, Arginine (Arg); 11, γ-amino-butyrate (GABA); 12, Acetate (AC); 13, N-acetyl-aspartic acid (NAA); 13, N-acetyl-glutamate (NAG); 15, Methionine (Met); 16, Glutamate (Glu); 17, Glutathione (GSH); 18, Acetoacetate (Ace); 19, Pyruvate (Pyr); 20, Succinate (Suc); 21, Glutamine (Gln); 22, Asparate (Asp); 23, Citrate (Cit); 24, Isocitrate (Isoc); 25, Malate (Mal); 26, Trimethylamine (TMA); 27, Creatinine (Cre); 28, Creatine (Cr); 29, Phosphocreatine (PCr); 30, Ethanolamine (ETA); 31, choline (Cho); 32, O-phosphocholine (OPC); 33, Taurine (Tau); 34, Betaine (Bet); 35, Myo-inositol (Myo); 36, Methanol (MeOH); 37, Glycine (Gly); 38, Glycerol (Gyo); 39, Ascorbate (Asc); 40, Serine (Ser); 41, Inosine (Ino); 42, Uracil (Ura); 43, Uridine (UDP); 44, Adenosine (Ade); 45, Adenosine monophosphate monophosphate (AMP); 46, Fumarate (Fum); 47, Tyrosine (Tyr); 48, Histidine (His); 49, Tryptophan (Trp); 50, Phenylalanine (Phe); 51, Nicotinamide (Nict); 52, Nicorinurate (Nicr); 53, Xanthine (Xan); 54, 3-Methylxanthine (3-MX); 55, Hypoxanthine (Hyp).

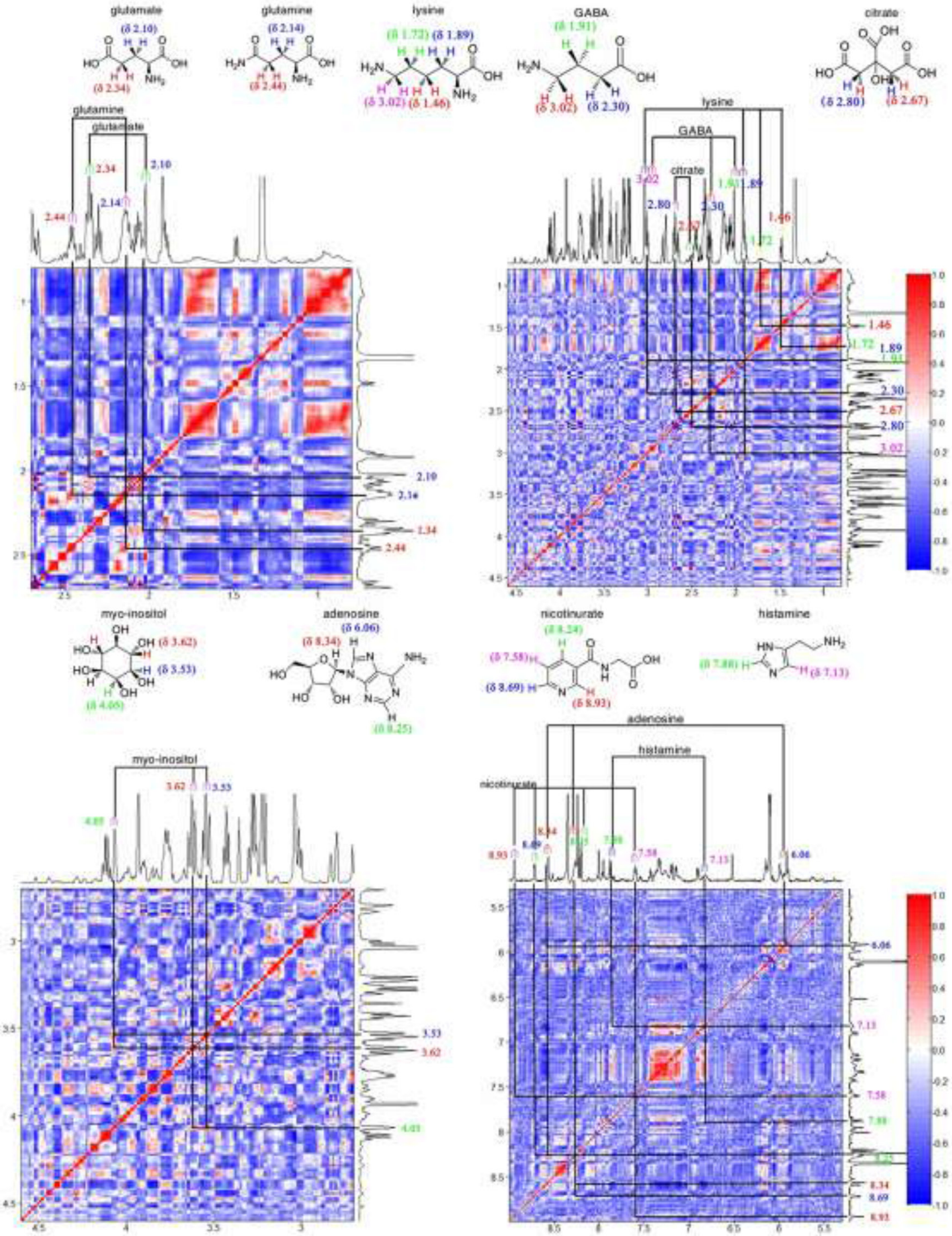

Supplementary Figure S3: Two-dimensional STOCSY image plots with assignment examples of some metabolites. The degree of correlation across the spectrum has been color coded and projected on the spectrum.

Supplementary Table S1: The synergistic experimental design of the HLJDD

Group	Berberine (mg/kg)	Baicalin (mg/kg)	Jasminoidin (mg/kg)	Dose (mg/kg)
ABC	8.6	6.8	4.6	20
A	20			20
B		20		20
C			20	20
AB	11.1	8.9		20
AC	13		7	20
BC		11.9	8.1	20

Supplementary Table S2: The assignment of metabolites in cerebrum of N, M and ABC rats.

See Supplementary File 1

Supplementary Table S3: The assignment of metabolites in serum of N, M and ABC rats.

See Supplementary File 2

Supplementary Table S4: Primers used for real-time PCR assays performed on the LC480 system

Genes	Forward primer (5’-3’)	Reverse primer (5’-3’)
NADPH	TGGCCAACGAAGGGGTTAAA	CACTGAGAAGTTCAGGGCGT
GSS	ACAACGAGCGAGTTGGGAT	TGAGGGGAAGAGCGTGAATG
HO-1	TGCACATCCGTGCAGAGAAT	CTGGGTTCTGCTTGTTTCGC
Nrf2	CATTTGTAGATGACCATGAGTCGC	GCCAAACTTGCTCCATGTCC
Bcl-2	TTTCTCTCTTTCGGCCGTGG	TATCCCACTCGTAGCCCCTC
Caspase 3	GGAGCTTGGAACGCGAAGA	ACACAAGCCCATTTCAGGGT
Bax	AGGACGCATCCACCAAGAAG	CAGTTGAAGTTGCCGTCTGC

NADPH: nicotinamide adenine dinucleotide phosphate; GSS: glutathione synthase
